# ITPase deficiency causes a Martsolf-like syndrome with a lethal infantile dilated cardiomyopathy

**DOI:** 10.1371/journal.pgen.1007605

**Published:** 2019-03-11

**Authors:** Mark T. Handley, Kaalak Reddy, Jimi Wills, Elisabeth Rosser, Archith Kamath, Mihail Halachev, Gavin Falkous, Denise Williams, Phillip Cox, Alison Meynert, Eleanor S. Raymond, Harris Morrison, Stephen Brown, Emma Allan, Irene Aligianis, Andrew P. Jackson, Bernard H. Ramsahoye, Alex von Kriegsheim, Robert W. Taylor, Andrew J. Finch, David R. FitzPatrick

**Affiliations:** 1 MRC Human Genetics Unit, Institute of Genomic and Molecular Medicine, University of Edinburgh, Edinburgh, United Kingdom; 2 Section of Genetics, Leeds Institute of Biomedical and Clinical Sciences, University of Leeds, Leeds, United Kigndom; 3 University of Florida College of Medicine, Center for NeuroGenetics, Gainesville, United States of America; 4 Edinburgh Cancer Research Centre, MRC Institute of Genomic and Molecular Medicine, University of Edinburgh, Edinburgh, United Kingdom; 5 Department of Clinical Genetics, Great Ormond St Hospital, London, United Kingdom; 6 Medical School, University of Oxford, John Radcliffe Hospital Oxford United Kingdom; 7 Wellcome Centre for Mitochondrial Research, Institute of Neuroscience, Newcastle University, Newcastle upon Tyne, United Kingdom; 8 Department of Clinical Genetics, Birmingham Women's and Children's NHSFT, Birmingham, United Kingdom; 9 Department of Histopathology, Birmingham Women's and Children's NHSFT, Birmingham United Kingdom; 10 CBS-IGMM Transgenic Unit, University of Edinburgh, Edinburgh, United Kingdom; 11 Centre for Genetic and Experimental Medicine, Institute of Genomic and Molecular Medicine, University of Edinburgh, Edinburgh, United Kingdom; University College London, UNITED KINGDOM

## Abstract

Typical Martsolf syndrome is characterized by congenital cataracts, postnatal microcephaly, developmental delay, hypotonia, short stature and biallelic hypomorphic mutations in either *RAB3GAP1* or *RAB3GAP2*. Genetic analysis of 85 unrelated “mutation negative” probands with Martsolf or Martsolf-like syndromes identified two individuals with different homozygous null mutations in *ITPA*, the gene encoding inosine triphosphate pyrophosphatase (ITPase). Both probands were from multiplex families with a consistent, lethal and highly distinctive disorder; a Martsolf-like syndrome with infantile-onset dilated cardiomyopathy. Severe ITPase-deficiency has been previously reported with infantile epileptic encephalopathy (MIM 616647). ITPase acts to prevent incorporation of inosine bases (rI/dI) into RNA and DNA. In *Itpa*-null cells dI was undetectable in genomic DNA. dI could be identified at a low level in mtDNA without detectable mitochondrial genome instability, mtDNA depletion or biochemical dysfunction of the mitochondria. rI accumulation was detectable in proband-derived lymphoblastoid RNA. In *Itpa*-null mouse embryos rI was detectable in the brain and kidney with the highest level seen in the embryonic heart (rI at 1 in 385 bases). Transcriptome and proteome analysis in mutant cells revealed no major differences with controls. The rate of transcription and the total amount of cellular RNA also appeared normal. rI accumulation in RNA–and by implication rI production—correlates with the severity of organ dysfunction in ITPase deficiency but the basis of the cellulopathy remains cryptic. While we cannot exclude cumulative minor effects, there are no major anomalies in the production, processing, stability and/or translation of mRNA.

## Author summary

Nucleotide triphosphate bases containing inosine, ITP and dITP, are continually produced within the cell as a consequence of various essential biosynthetic reactions. The enzyme inosine triphosphate pyrophosphatase (ITPase) scavenges ITP and dITP to prevent their incorporation into RNA and DNA. Here we describe two unrelated families with complete loss of ITPase function as a consequence of disruptive mutations affecting both alleles of *ITPA*, the gene that encodes this protein. Both of the families have a very distinctive and severe combination of clinical problems, most notably a failure of heart muscle that was lethal in infancy or early childhood. They also have features that are reminiscent of another rare genetic disorder affecting the brain and the eyes called Martsolf syndrome. We could not detect any evidence of dITP accumulation in double-stranded DNA from the nucleus in cells from the affected individuals. A low but detectable level of inosine was present in the circular double-stranded DNA present in mitochondria but this did not have any obvious detrimental effect. The inosine accumulation in RNA was detectable in the patient cells. We made both cellular and animal models that were completely deficient in ITPase. Using these reagents we could show that the highest level of inosine accumulation into RNA was seen in the embryonic mouse heart. In this tissue more than 1 in 400 bases in all RNA in the cell was inosine. In normal tissues inosine is almost undetectable using very sensitive assays. The inosine accumulation did not seem to be having a global effect on the balance of RNA molecules or proteins.

## Introduction

It is 40 years since two brothers were reported with severely delayed neurocognitive development, spasticity, postnatal microcephaly, short stature, congenital cataracts and primary hypogonadism[1], characterising a disorder that is now termed Martsolf syndrome (MIM 212720). Warburg Micro syndrome (MIM 600118, 614225, 615222, 615663) is an overlapping condition that was described in 1993, which also has microphthalmia/microcornea, retinal dystrophy, optic nerve atrophy and intracranial malformations as clinical features[2]. 60% of cases referred to us with a diagnosis of Warburg Micro syndrome have loss-of-function mutations in either *RAB3GAP1*, *RAB3GAP2*, *RAB18* or *TBC1D20*[3–6]. 44% of Martsolf syndrome cases have mutations in *RAB3GAP1* or *RAB3GAP2*, which perturb but do not completely abolish the expression or function of the encoded protein[7, 8]. The relatively high proportion of unexplained cases in both syndromes indicates that there are likely to be more disease loci and/or causative genetic mechanisms to be discovered.

Infantile-onset dilated cardiomyopathy (iDCM) is a rare, aetiologically heterogeneous disorder that may present as acute, commonly lethal, and with cardiogenic shock [9]. Isolated iDCM may be caused by genetically determined primary abnormalities of heart muscle (sarcomere, Z-disc, desmosomes etc) while iDCM as a component of a multisystem disorder is most commonly secondary to an inborn error of metabolism (**Table 1**)[10–12] with the prognosis being dependent on the underlying cause. Early genetic testing is recommended in iDCM as it may help direct the clinical management[13].

**Table 1:** Genetic causes of syndromic infantile-onset dilated cardiomyopathy.

Name of Disorder	OMIM	Genes	Progressive Neurological Disorder	Eye Disease	Other Features	References
**Alstrom Syndrome**	#203800	*ALMS1*	No	Yes	AR. Systemic fibrosis, multiple organ involvement, retinal degeneration, hearing loss, childhood obesity, diabetes mellitus, urogenital dysfunction. Pulmonary, hepatic and renal failure.	[39]
**Barth Syndrome**	#302060	*TAZ*	No	No	X-linked recessive. Neutropenia, growth delay and skeletal myopathy. Gross motor delay, early death from septicaemia. Intermittent lactic acidaemia, muscle weakness. Endocardial fibroelastosis.	[40–42]
**Barth-like condition (3-Methylglutaconic aciduria Type V)**	#610198	*DNAJC19*	+/-	No	AR. Prenatal or postnatal growth failure, cerebellar ataxia, significant motor delay, testicular dysgenesis-cryptorchidism, severe perineal hypospadias, elevated plasma and urine 3-methylglutaconic acid and 3-methylglutaric acid.	[43, 44]
**Carnitine Palmitoyltransferase II Deficiency**	#600649	*CPT2*	No	No	AR. Recurrent hypoglycaemia, seizures, liver failure and transient hepatomegaly. Episodes triggered by infections, fever, or fasting. Elevated creatine kinase.	[45, 46]
**Carvajal Syndrome**	#605676	*DSP*	No	No	AR. Cardiocutaneous syndrome: woolly hair, palmoplantar keratoderma, tooth agenesis.	[47]
**Familial neonatal isolated cardiomyopathy**	#613642	*SDHA*	No	No	Normal growth and neuromuscular examination. Normal development, no seizures. Normal brain MRI.	[48]
**Long-chain 3-hydroxyl CoA Dehydrogenase Deficiency**	#609016	*HADHA*	Yes	Yes	AR. Coma, hypoglycaemia, myopathy, hepatopathy, intellectual disability, infantile spasms (-/+). Brain imaging: atrophic changes to parieto-occipital lesions, abnormal EEG background. Choreo-retinal changes, abnormal ERG.	[49–51]
**Primary Carnitine Deficiency**	#212140	*SLC22A5*	Yes	No	AR. Hypoketotic hypoglycaemia (encephalopathy), hepatomegaly, muscle weakness, cardiac arrhythmia, asymptomatic to sudden death.	[52, 53]

Here we report two families with a very distinctive clinical presentation of lethal iDCM and Martsolf-like syndrome associated with homozygous null mutations in *ITPA* which encodes inosine triphosphate pyrophosphatase (ITPase). ITPase is an enzyme that functions to prevent incorporation of inosine bases (rI/dI) into RNA and DNA by scavenging ITP/dITP in the cell. An autosomal recessive partial deficiency of inosine triphosphate pyrophosphatase (ITPase) has been recognised since the late 1960’s via accumulation of inosine triphosphate (ITP) in erythrocytes[14]. This is a relatively common trait that is clinically asymptomatic although it may influence sensitivity to certain drugs[15]. The trait is caused by hypomorphic mutations in *ITPA* (the gene encoding ITPase) which affect splicing and/or protein stability[16]. Biallelic loss-of-function mutations in *ITPA* have recently been reported as the cause of an early infantile encephalopathy (EIEE35, MIM #616647)[17]. We present data testing and refuting various hypotheses (summarised below in Fig 6) regarding the molecular consequences of ITPase deficiency on the genome, transcriptome and proteome.

## Results

### Clinical information

**Fig 1:**
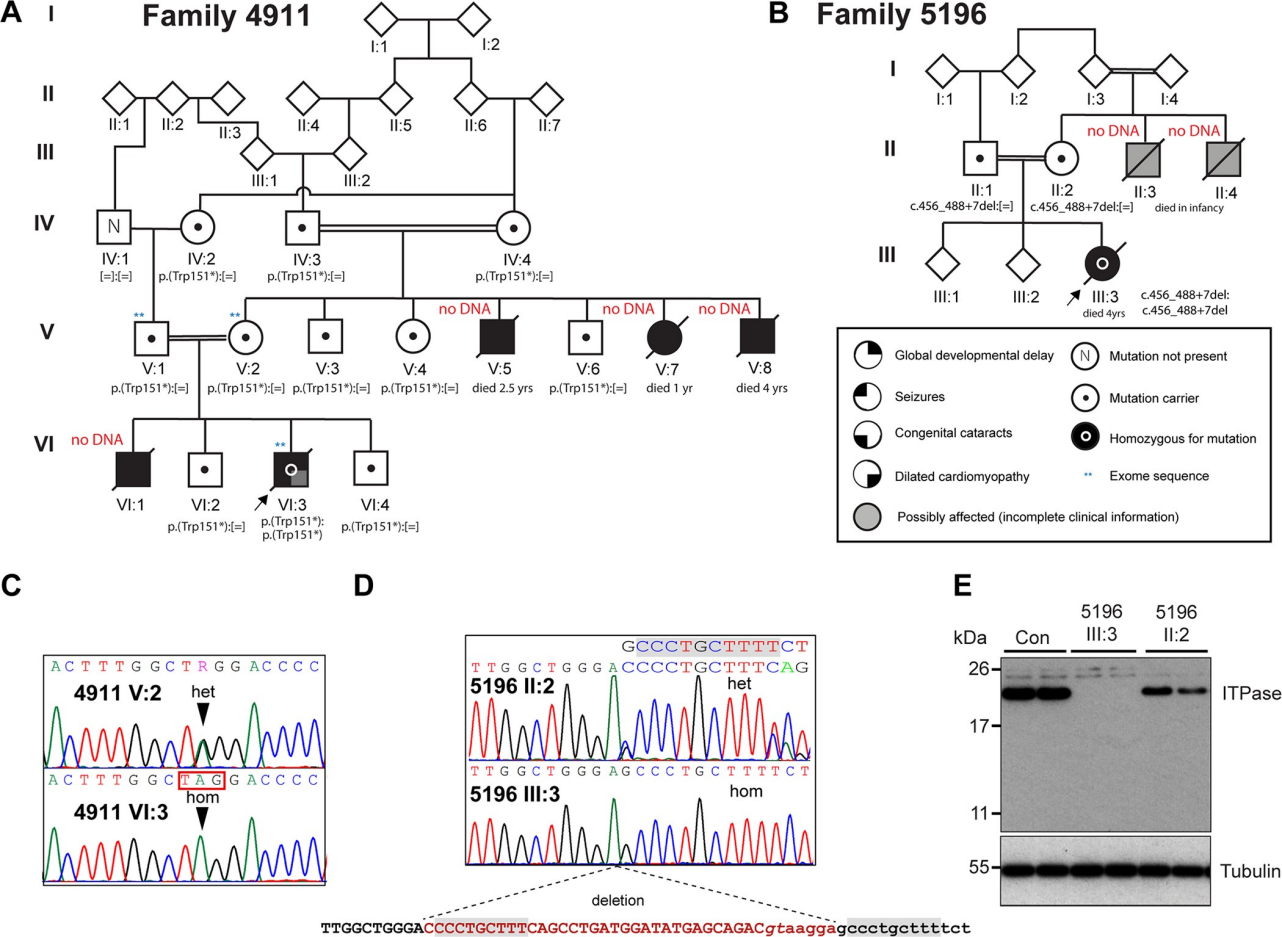
Loss-of-function mutations in *ITPA* identified in Martsolf-like syndrome with infantile cardiomyopathy. (A) Pedigree for Family 4911 showing the transmission of the *ITPA* c.452G>A, p.Trp151* allele. (B) Pedigree for Family 5196 showing the transmission of the *ITPA* c.456_488+7del allele. Electropherograms from sequencing of the affected individual (lower) and his mother (upper) from Family 4911 (C) and the affected individual (lower) and her mother (upper) from Family 5196 (D) show the sequence changes. The mutation nomenclature is based on the reference sequences NM_033453 and NP_258412. (E) Western blotting shows that ITPA protein is absent in a lymphoblastoid cell line derived from the affected individual 5196 III:3 and reduced in a line derived from her mother. Blotting for Tubulin serves as a loading control and each lane on the blot corresponds to an individual lysate sample.

In Family 4911 (**Fig 1A**) a maternal uncle (4911 V:5) and aunt (4911 V:7) of the proband (VI:3) had been described in a clinical paper as Martsolf syndrome with a previously unreported association with an early-onset cardiomyopathy[18]. The proband in the present study, their nephew, died at the age of 2 years. No post mortem examination was carried out and the exact cause of his death could not be confirmed. Prior to his demise he had been noted to have postnatal onset microcephaly with severe delay in all aspects of his development. He had bilateral cataracts diagnosed at the age of 13 months. Generalised seizures began at the age of 14 months. He was noted to have small genitalia. A clinical diagnosis of Martsolf syndrome was made and he had a negative screen for *RAB3GAP1*, *RAB3GAP2*, *RAB18* and *TBC1D20*. His elder brother, two maternal uncles and a maternal aunt all had a very similar pattern of problems (**Table 2**) and all had died in early childhood with evidence of cardiac failure[18]. Serial echocardiograms in 4911 VI:3 had shown persistent but mild dilation of his left ventricle and he is assumed to have died as a result of the progression of his cardiac disease.

**Table 2:** Clinical features.

	Family 4911					Family 5196
	V:5	V:7	V:8	VI:1	VI:3	III:3
Ethnicity	Pakistan					Pakistan
Consanguinity	Yes					Yes
Family history	See Pedigree Fig 1A					See Pedigree Fig 1B
Sex	Male	Female	Male	Male	Male	Female
Age at last assesment	9 mo	9 mo		not seen	15 months	4 yrs
Age at death	2.5 years	1yr	4yr	6 months	2yrs	4yrs
**Birth and newborn**						
Gestation	40	40	37	?	40	39
Birth weight g [z score]	3200 [-0.71]	2800	2070	?	?	2892
**Developmental milestones**						
Social smile		>9 months	?	?	?	8 months
Sat Independently						Not achieved
Walked Independently	Not achieved	Not achieved	Not achieved	Not applicable	Not achieved	Not achieved
First words	Not achieved	Not achieved	Not achieved	Not applicable	Not achieved	Not achieved
**Growth parameters**						
Height/length cm [z score]	71 [-0.33]	67 [-1.3]	?	?	?	97 [-1.1]
Weight kg [z score]	7.7 [-1.7]	6.5 [2.7]	?	?	?	10.2 [-4.2]
OFC cm [z score]	40 [-5.4]	39.7 [-4.8]	?	?	?	42 [-7.6]
**Clinical features**						
Neurology	Hypotonic, brisk reflexes	Hypotonic, brisk reflexes, poor bulbar function	Hypotonia. OFC< 3rd centile. Optic atrophy	Delayed	Hypotonia. Developmental delay	Hypotonia, Severe developmental delay, microcephaly
Seizures	None	Started 8 months, facial twitching		None	started 14 months, generalised	Presented at 5 months with status epilepticus
EEG		Multifocal spikes and sharp waves	bursts of irregular activity		normal	normal
Neuroimaging	CT Brain showed generalised cerebral atrophy	CT and MRI of brain; generalised cerebral and brainstem atrophy	None	None	None	Neuropathology: see Fig 2
Cardiac	Cardiac failure	Dilated cardiomyopathy	Dilated cardiomyopathy	Presented in cardiac failure at 6 months of age. Died suddenly before formal assessment	Mild dilation of left ventricle	See Fig 2
Eye/vision	Central lens opacities	Bilateral Cataracts	Bilateral Cataracts	Bilateral cataracts. Developed between 2 and 6 months of age	Bilateral cataracts detected 13 months	Bilateral cataracts
Additional malformations	Cryptorchidism, slight hirsuitism	Slight hirsuitism	Microcephaly. Cryptorchidism		Small genitalia, microcephaly	Severe thymic atrophy, gracile bones on skeletal survey
**ITPA variants (GRCh37/hg19)**	No DNA available	No DNA available	No DNA available	No DNA available	chr20 g.3202527G>A; c.452G>A, p.Trp151* (rs200086262); homozygous	chr20 hg19 g.3202531-3202570del; c.456_488+7del; homozygous

**Fig 2:**
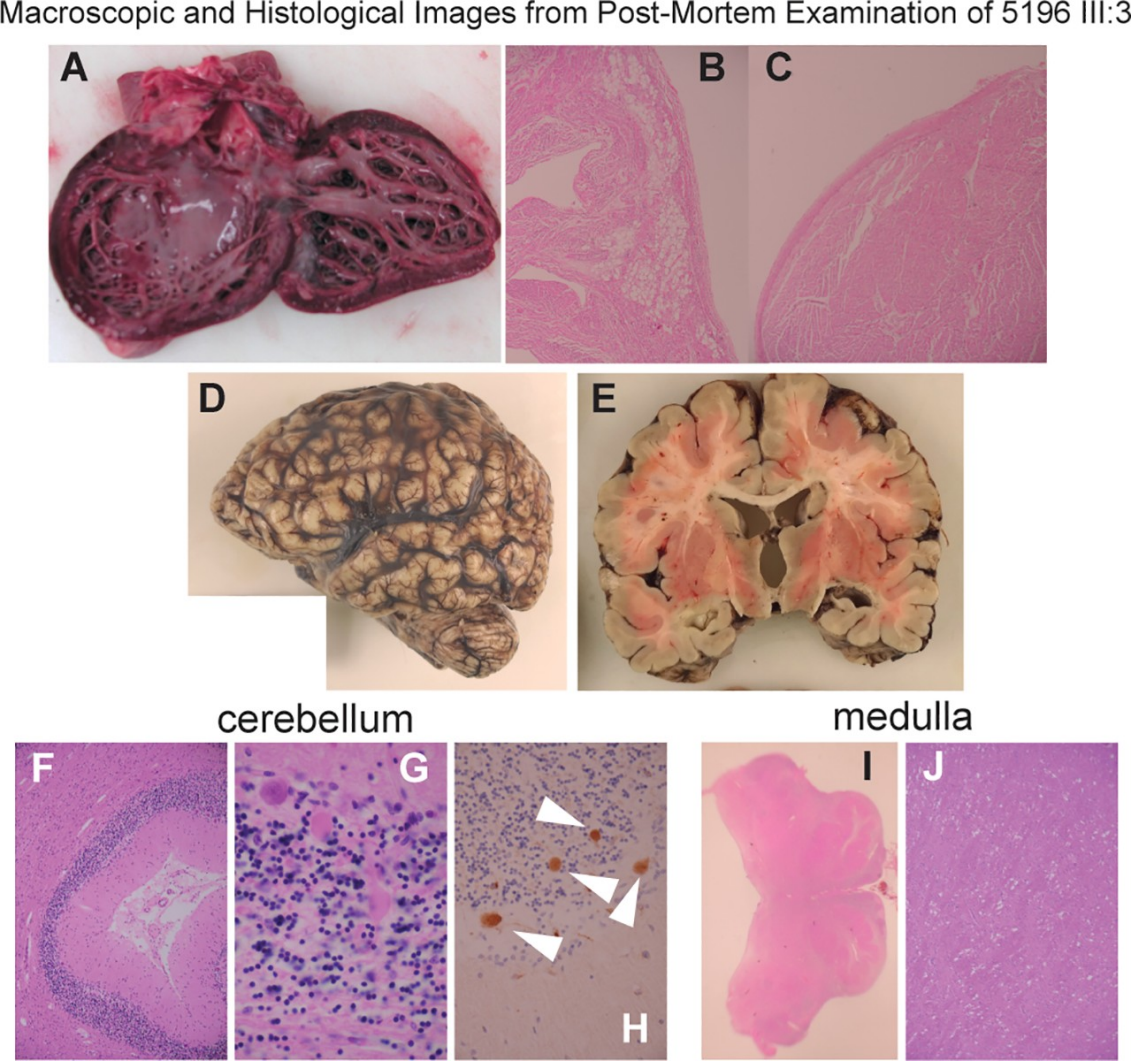
Phenotype associated with complete loss of ITPase function. (A) Photograph of the dissected heart from the affected individual (III:3) in Family 5196 showing increased trabeculation and fibroelastosis of the endocardium. (B,C) Photomicrographs (x200) showing fatty infiltration and fibroelastosis of the heart respectively (D) Photograph of the fixed brain showing microcephaly/micrencephaly. (E) Coronal slice through brain showing dilatation of the lateral and third ventricles with reduced volume of white matter. (F) Image (x100) showing atrophy of the cerebellar cortex. (G) Photomicrograph (x600) shows degenerate Purkinje cells in the cerebellar cortex with spheroids (white arrows) in the B-APP immunostain (H, x400). (I) Macroscope (x4) image of the medulla showing hypoplastic pyramidal tracts. (J) Photomicrograph (x100) showing vacuolation of the pyramidal tracts.

In family 5196 (**Fig 1B**) the affected proband (5196 III:3) was a girl who died at the age of 4 years. She had previously been clinically diagnosed by an experienced clinical geneticist as having Martsolf syndrome on the basis of profound developmental delay, failure to thrive, microcephaly, seizures and congenital cataracts. Screening of the known Martsolf syndrome and Warburg Micro genes was negative. She presented in severe cardiac failure and died shortly after this. She had not previously been suspected of having any cardiac disease. In addition to the known anomalies, a post mortem examination revealed marked dilation of the left ventricle with increased trabeculation and mild fibroelastosis (**Fig 2A–2C**). Fatty infiltration was noted of the right ventricle (**Fig 2B**). Neuropathology showed cerebellar atrophy (**Fig 2F**), microgliosis of dentate and olivary nuclei, vacuolation of white matter (**Fig 2E**) with scattered axonal spheroids (**Fig 2H**) and gliosis of the hippocampus. 5196 III:3 had two maternal uncles who died in infancy (5196 II:3 and 5196 II:4; **Fig 1B**) who were suspected of having the same disorder although no clinical details were available from either individual.

### Whole exome sequencing and cohort resequencing

In an effort to identify additional causative genes for Martsolf and Martsolf-like syndromes we used whole-exome sequencing (WES) of affected individuals from consanguineous multiplex families (Family 4911 VI:3; Family 5196 III:3) and their healthy parents (4911 V:1 & 4911 V:2; 5196 II:1 & 5196 II:2) (**Fig 1**). These UK families had no known shared relatives but both had recent Pakistani ancestry. The alignment, read depth and estimated heterozygous SNP detection sensitivity of each individual is given in **S1 Table**[19]. Sequence variants in the probands were filtered using minor allele frequency (MAF) of < 0.001, plausibly deleterious consequence and bi-allelic inheritance. The WES data from the unaffected parents was used primarily to confirm biallelic inheritance in their children and to exclude shared homozygous variants. The percentage coverage of the WES capture sequences >15X for the unaffected parents 4911 V:1 and 4911 V:2 was significantly lower than expected (S1 Table) but was sufficient for analysis of the candidate high impact variants in their affected son 4911 VI:3.

In 4911 VI:3, ten rare homozygous variants (**Table 3**) were reviewed by manual assessment of read quality using IGV2.3 software[20] and by Sanger sequencing of selected variants for their segregation in the family. Of these only a nonsense mutation, c.452G>A, p.Trp151* (rs200086262) in *ITPA* (NM_033453, MIM 147520) segregated within the family in a manner consistent for an autosomal recessive disease-causing mutation (**Fig 1A**). This variant has been previously identified as disease associated[17] and is present in gnomAD (genome Aggregation Database) with a minor allele frequency of 0.0058%.

**Table 3:** Rare homozygous variants detected in proband 4911 VI:3.

Chromosme	Position	Reference nucleotide	Alternative nucleotide	rsID	Gene Name	Transcript ID	Peptide change	Segregation
**1**	220267581	G	T	rs149324758	*IARS2*	ENST00000302637	Arg8Leu	No
**2**	10192477	G	A	rs199770737	*KLF11*	ENST00000305883	Arg461Gln	No
**9**	114246291	C	T		*KIAA0368*	ENST00000259335	Gly177Glu	No
**13**	42761244	C	A	rs147914294	*DGKH*	ENST00000536612	Thr397Asn	No
**17**	56783857	G	A		*RAD51C*	ENST00000425173	Cys170Tyr	No
**18**	5478295	C	A	rs117900256	*EPB41L3*	ENST00000341928	Ser109Ile	No
**19**	1918914	C	T		*SCAMP4*	ENST00000316097	Ala107Val	No
**19**	35524607	G	A	rs72558029	*SCN1B*	ENST00000262631	Val138Ile	No
**20**	3202527	G	A	rs200086262	*ITPA*	ENST00000380113	Trp151*	Yes
**22**	51039276	T	G		*MAPK8IP2*	ENST00000329492	Leu10Arg	No

In the affected individual from Family 5196 (III:3), homozygosity for an apparently unique 40bp deletion spanning the splice donor site of *ITPA* exon 7 was detected on WES. Subsequent Sanger sequencing confirmed this to be chr20 hg19 g.3202531-3202570del; c.456_488+7del. This deletion is likely to have been microhomology-mediated as a nine base pair perfect repeat is present at the 5’ end of the deleted region and the genomic region immediately 3’ to the breakpoint (**Fig 1D**). Both parents (5196 II:1 and 5196 II:2) were heterozygous for this mutation. Western blotting of lysates from lymphoblastoid cell lines (LCLs) from 5196 III:3 and her mother showed that ITPA protein was completely absent in the cells derived from the affected girl (**Fig 1E**). We were not able to identify other plausibly causative genotypes in 5196 III:3 in any known developmental disease genes using our previously described DDG2P diagnostic pipeline [21]

Sanger sequencing of *ITPA* in the remaining members of the cohort of 85 “mutation negative” families[8] revealed no further plausibly disease-associated mutations. The primers used for this analysis are given in **S2 Table**.

### Inosine ribobases (rI) are incorporated in RNA from an affected individual

**Fig 3:**
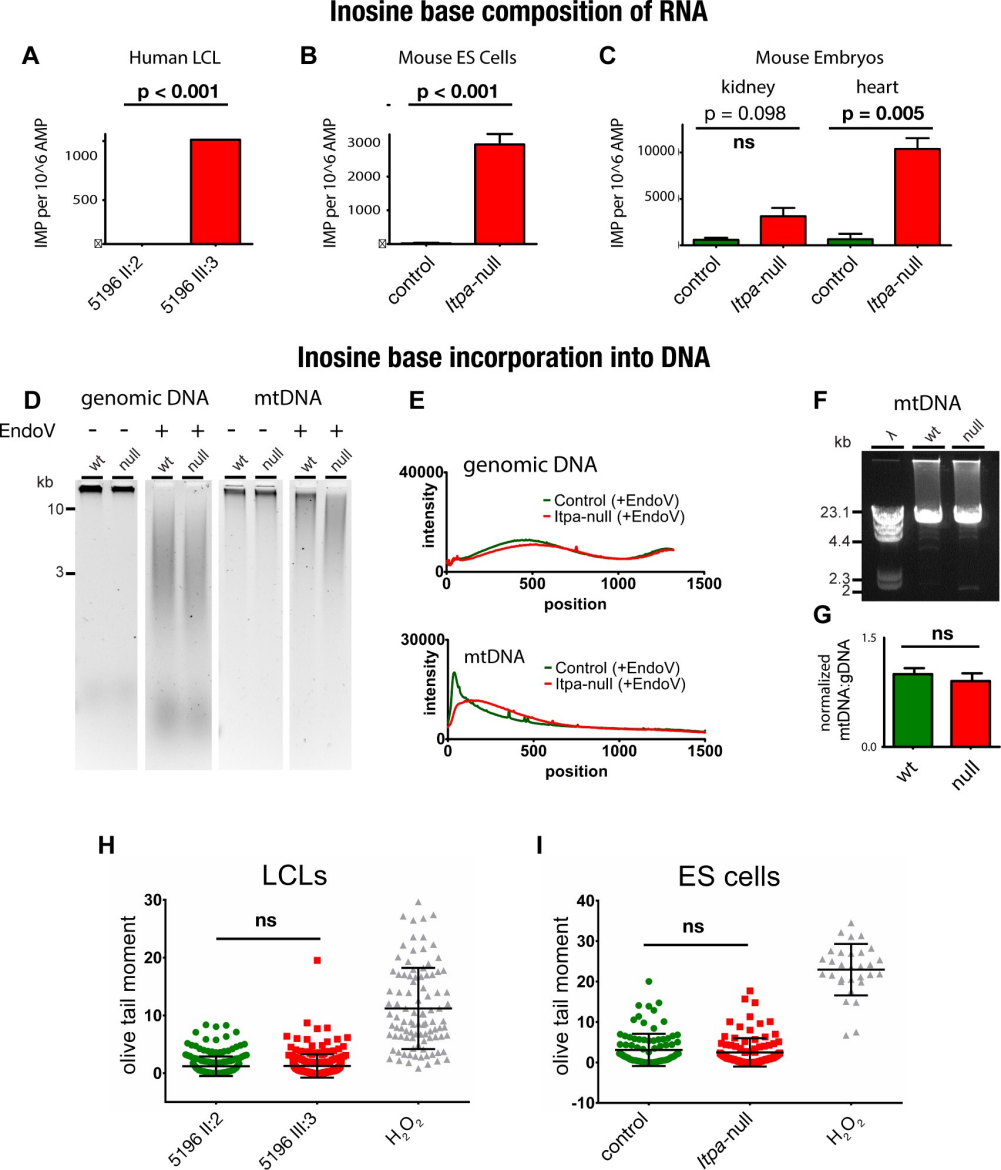
Inosine incorporation into nucleic acids in human and mouse cells lacking functional ITPase. **(A)** Bar chart showing a significantly increased inosine base content of RNA in lymphoblastoid cell lines (LCLs) derived from an affected individual (5196 III:3) as compared to that derived from her mother (5196 II:2) **(B)** Bar chart showing significantly increased inosine base content of RNA in *Itpa*-null mouse embryonic stem (ES) cells as compared to control ES cells. **(C)** Bar chart showing increased inosine base content of RNA derived from *Itpa*-null tissue as compared to controls. Inosine content is significantly higher in RNA derived from *Itpa*-null hearts than that stage-matched control hearts. There was no significant (ns) difference in IMP content in RNA derived from *Itpa*-null compared to control kidneys. Error bars ±SEM. **(D)** Alkaline-gel electrophoresis of total DNA and mtDNA extracted from mouse ES cells untreated or treated with bacterial endonuclease V (EndoV). All lanes shown are on the same gel, and these data are representative of three independent experiments. **(E)** Densitometry of gels shown in D does not identify any difference between control (green lines) and *Itpa*-null (red lines) cells for genomic DNA (top panel) but for mtDNA (bottom panel) there is a shift in the migration pattern in the *Itpa*-null cells suggestive of an increase EndoV digestion compared to the controls. **(F)** Long-range PCR (LR-PCR) of the mitochondrial genome shows no evidence for increased deletions in *Itpa*-null ES cells as compared to controls. The data shown are representative of three independent experiments. The primers used are listed in **S2 Table**. **(G)** Quantitative RT-PCR (qPCR) on total DNA shows that ratios of mtDNA to genomic DNA are comparable between control and *Itpa*-null cells. The data shown are derived from analysis of six individual DNA preparations per genotype, each analysed in triplicate. All the primers used are listed in **S2 Table**. **(H,I)** Alkaline comet assays on LCLs derived from an affected individual (5196 III:3) and her mother (5196 II:2) and null and parental mouse ESC respectively with cells exposed to hydrogen peroxide as a positive control. Neither cell type shows evidence for increase single or double strand breaks in genomic DNA. Quantitation of DNA damage is by Olive tail moment (the product of the tail length and the fraction of total DNA in the tail) and is a measure of both the extent of DNA fragmentation and size of fragmented DNA.

*ITPA* encodes inosine triphosphate pyrophosphatase (ITPase) which hydrolyzes both inosine triphosphate (rI) and deoxyinosine triphosphate (dI)[22, 23]. Its major function is thought to be to ensure the exclusion of these “non-canonical” purines from RNA and DNA in order to avoid transcript and genome instability. Complete deficiency of ITPase in all tissues would thus be predicted to result in an increase in the incorporation of rI and dI into RNA and DNA respectively. To test this we first purified cellular RNA from a lymphoblastoid cell line (LCLs) from 5196 III:3. This RNA was digested to single nucleotides and analysed using a combination of HPLC and mass spectrometry (HPLC/MS). Using this approach we found that rI was present in RNA at a level of 725±158 SEM nucleotides of rI per 10^6^ nucleotides of AMP in 5196 III:3, a significantly higher level than in RNA from LCLs derived from either her father (17±11 SEM rI:rA x 10^6^) or her mother (71±60 SEM rI:rA x 10^6^) (**Fig 3A**). This equates to approximately one rI base in every 5500 bases of RNA from the null LCL.

### Inosine ribobases (rI) are incorporated in RNA from *Itpa*-null mouse ES cells and embryonic tissues

**Fig 4:**
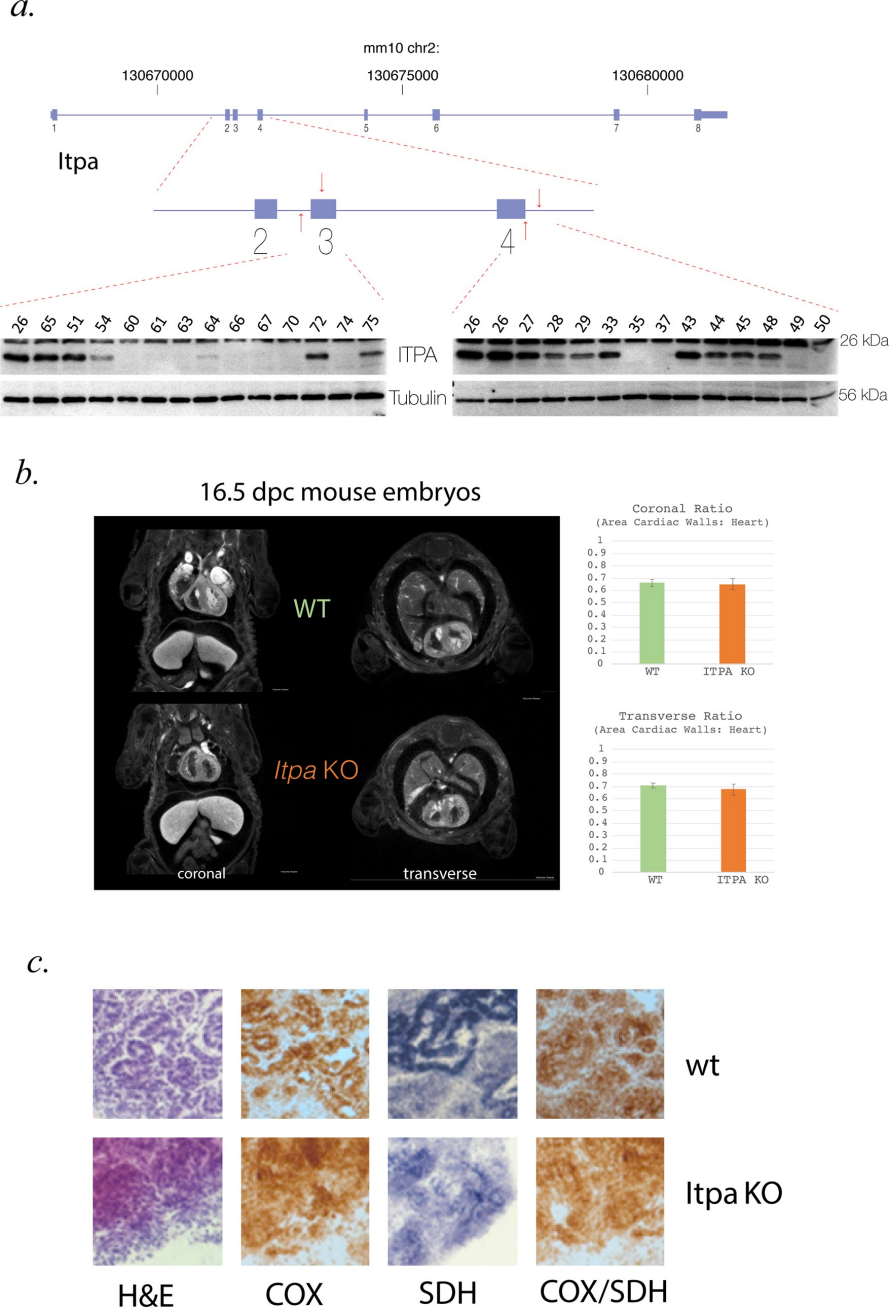
Creation, morphology and biochemical analysis of *Itpa* null mouse embryos. **(A)** Cartoon representation of the mouse *Itpa* genomic locus and gene structure with a more detailed diagram of exons 2–4 indicating the position of the guideRNAs used to create the null alleles in the mouse lines to create null embryos. Representative western blots are shown of embryonic tissue demonstrating absence of Itpa protein in samples used as “Itpa null”. ITPA protein is detected in lysates from control but not *Itpa*-null cells upon probing the blot with polyclonal antibodies raised to full-length ITPA (Millipore) and an N-terminal domain of the protein encoded by sequence 5’ of that mutated by CRISPR (LSBio). Blotting for Tubulin serves as a loading control and each lane on the blot corresponds to an individual lysate sample. **(B)** Representative coronal and transverse images through the heart from optical projection tomography (OPT) of wild-type (top panel) and *Itpa*-null (bottom panel) e16.5 embryos. The bar charts to the right of this image shows quantification of the heart wall to total heart area ratio which showed no difference between null (orange) and control (green) embryos. **(C)** Oxidative enzyme histochemistry of wild-type and *Itpa*-null embryonic heart. Sections were subjected to H&E staining, individual COX and SDH reactions together with sequential COX/SDH histochemistry. No evidence of morphological changes or focal enzyme deficiency in the *Itpa*-null heart was identified. Data are representative of duplicate experiments.

We generated *Itpa*-null mouse embryonic stem (ES) cells using CRISPR/Cas9 genome editing [24, 25] (**Fig 4A**, primers encoding the guide RNAs are detailed in **S2 Table**). In these cells, rI was detectable in RNA at 1889 nucleotides rI per 10^6^ nucleotides AMP ± 295 SEM (**Fig 3B**).

To determine if there was a correlation between the level of rI in different tissues *in vivo* and the organs affected in the human disease, we generated mice heterozygous for *Itpa* null alleles using direct cytoplasmic injection of Cas9 mRNA and guide RNAs into zygotes. Heterozygous animals were crossed to generate *Itpa*-null embryos and wild-type littermate embryos. Both genotyping and Western blot analysis (**Fig 4A**) were used to confirm the null status of each embryo (**Fig 4A**). As in previously reported targeted inactivation of *Itpa*, we found reduced body size (with a proportionate reduction in heart size) in *Itpa-*null embryos[26] and no other obvious morphological differences compared to wild-type controls (**Fig 4B**). The level of rI in RNA from *Itpa*-null hearts (10382 nucleotides IMP per 10^6^ nucleotides AMP ± 2008 SD) was significantly higher than in either *Itpa*-null brain or kidney (p<0.05 and p<0.01 respectively, student’s t-test) and equated to approximately one rI for every 385 bases of RNA (**Fig 3C**). rI was present at very low levels in RNA derived from control tissues.

### Inosine base incorporation is detectable in mtDNA but not genomic DNA

The bacterial endonuclease V (nfi/EndoV) cleaves DNA at dI bases creating nicks in the dsDNA[27]. Digestion of genomic DNA from control and *Itpa*-null ES cell lines using EndoV (New England Biolabs) followed by alkaline-gel electrophoresis revealed no measurable difference in migration between the samples (**Fig 3D**). However, a small but reproducible increase in the EndoV sensitivity of mtDNA from *Itpa*-null ES cells as compared to that in controls was seen (**Fig 3E**).

To assess whether this increased inosine incorporation was associated with increased instability of the mitochondrial genome (mtDNA), we used quantitative PCR (qPCR) to compare levels of mtDNA to levels of nuclear DNA. We also used long-range PCR (LR-PCR) of mtDNA to look for any increase in the frequency of mtDNA rearrangements. Neither assay showed any differences between *Itpa*-null cells and controls (**Fig 3F and 3G**), or between *Itpa*-null tissues and controls (**S1A and S1B Fig**). We used Ion Torrent sequencing to detect base substitutions and MinION sequencing to detect large-scale mtDNA rearrangements amplified from control and *Itpa*-null kidneys and hearts. No differences between wild type and *Itpa*-null kidney or heart were detected (**S1C and S1D Fig**).

To assess secondary effects of low-level dI incorporation on genome stability, a commercial comet assay kit (Trevigen) was used. No increase in DNA strand breaks could be detected in 5196 III:3 compared to 5196 II:2 LCLs, or in *Itpa*-null compared to wild-type ES cells (**Fig 3H and 3I**).

### Biochemical assessment of mitochondrial function

To assess whether ITPase deficiency had any effect on mitochondrial function, we carried out metabolic tracer analysis on the ES cells using ^13^C_5_-glutamine, and conducted functional histopathology on tissue samples. In the tracer experiments, ^13^C-incorporation into fumarate and citrate was analysed (**S1E Fig**). The (m+4) isotopologues of both metabolites indicated that ITPA-loss did not affect normal oxidative TCA cycle function and a low level of reductive carboxylation of oxoglutarate, as measured by citrate (m+5), was again minimally altered upon loss of ITPA. Functional histopathology on the tissue samples was adapted from analyses used in a clinical diagnostic setting. Samples were reacted for cytochrome *c* oxidase (COX) and succinate dehydrogenase (SDH) activities, with sequential COX-SDH histocytochemical analyses carried out in order to identify low level, focal COX-deficiency. No differences were seen between control and *Itpa*-null tissues and no COX-deficient cells were identified in *Itpa*-null heart (**Fig 4C**).

### Transcriptome and proteome analysis of *Itpa* null cells/tissues

**Fig 5:**
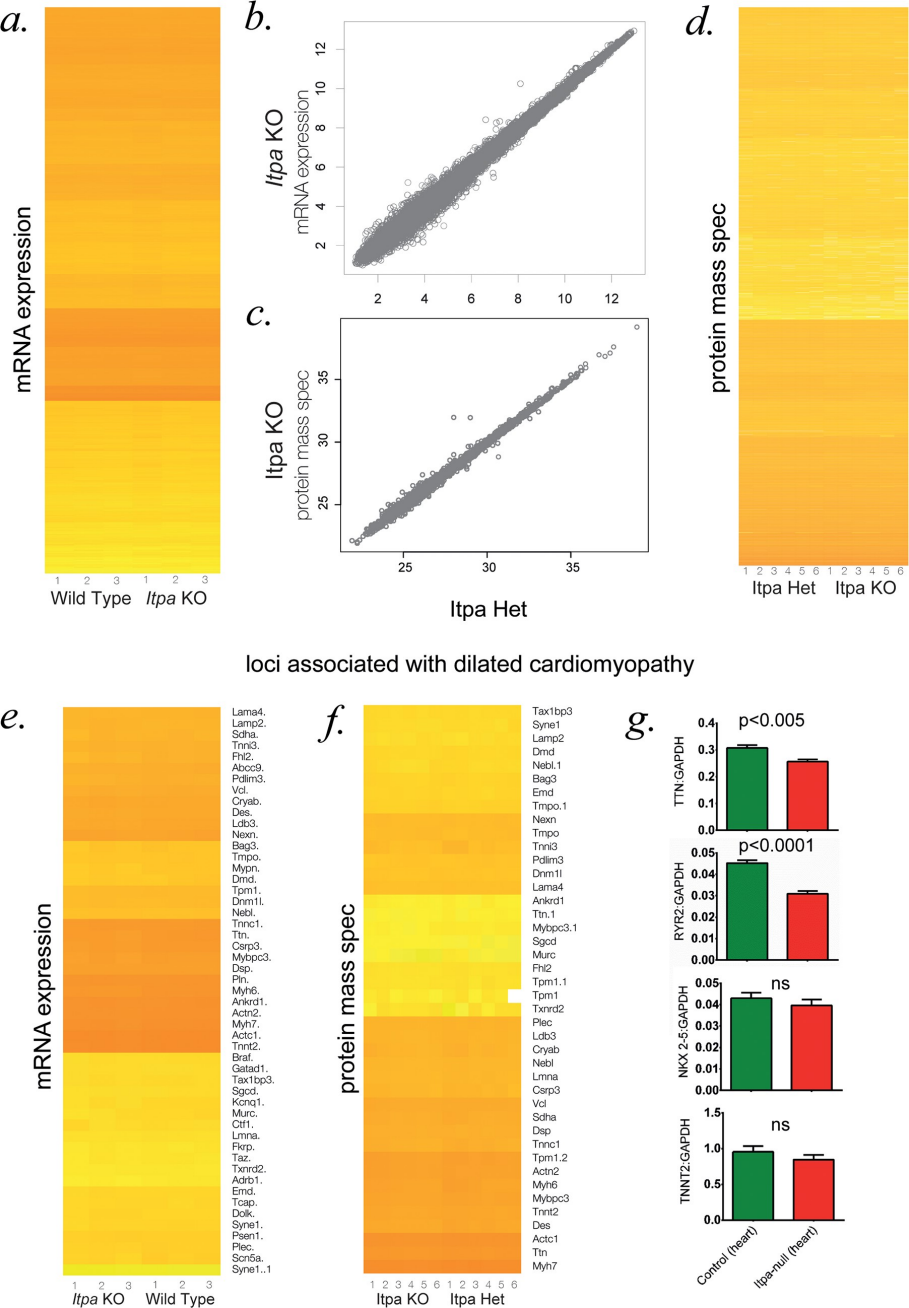
Transcriptomic and proteomic analyses of *Itpa* null heart. **(A)** Heatmap.2 based clustering of per-transcript genome wide log_2_ ratios from biological triplicates of RNAseq from *Itpa*-null embryonic hearts and littermate controls. **(B,C)** Plot of per-gene log_2_ signal from Affymetrix MTA1.0 microarray (same data as A) and quantitative protein mass spectrometry (same data as D) on samples from wild-type and *Itpa*-null embryonic hearts. **(D)** Heatmap.2 based clustering of genome wide per-protein intensity from six biological replicates of quantitative mass spectrometry from *Itpa*-null embryonic hearts and littermate controls. **(E, F)** Subset of data from A & D focussed on transcripts and proteins that are known to be involved in mendialian causes of dilated cardiomyopathy. No major differences are detectable in any of the heatmaps. **(G)** Quantitative RT-PCR (qPCR) of selected transcripts in *Itpa*-null embryonic hearts and littermate controls. The data shown are derived from analysis of 10 individual cDNA preparations per genotype, each analysed in triplicate.

We compared the transcriptome of control and *Itpa*-null mouse hearts using the Affymetrix MTA1.0 expression microarray. There was very strong concordance between transcript levels in control and *Itpa*-null samples when all loci were examined together (**Fig 5A and 5B**) or when a subset of loci that have been implicated in dilated cardiomyopathy in mice or humans were examined separately (**Fig 5E**). When specific cardiac disease genes were examined using ddPCR, modest reductions could be observed in *Itpa*-null heart tissue (**Fig 5G**). However it was not possible to determine if reductions of this magnitude would significantly alter cardiomyocyte function and, more importantly, we could not distinguish whether these changes in transcript levels were primary effects or secondary to an early disease process in heart.

A generalized effect on transcription produced by increased inosine incorporation into RNA in *Itpa*-null cells might not be identified on transcriptome analysis if its effects on individual transcripts are proportionate. Therefore, in order to assess any changes in transcription rate or transcript stability, we labelled RNA transcribed over the course of 30 minutes with the ribonucleotide analogue 4-thiouridine (4sU). 4sU incorporation was assayed immediately after labelling, providing a measure of transcription rate, and then at subsequent time points, at which any changes in the rate of RNA turnover would be revealed (**S2A Fig**). 4sU incorporation was assayed by biotinylation of its thiol group and then quantification of biotin using a fluorescence-based kit. When RNA was harvested immediately following treatment, 4sU incorporation appeared lower in *Itpa*-null cells than in control cells. However, reduced incorporation was not seen when an alternative assay utilizing 5-Ethynyl Uridine was used (**S2C Fig**). No differences in the 4sU content of control and *Itpa*-null cells were observed at the later time points, suggesting that inosine incorporation does not affect RNA stability globally (**S2A and S2B Fig**).

Label-free quantitative mass spectrometry was performed to examine the whole proteome in control and *Itpa*-null mouse heart tissue. Apart from the absence of ITPase, no significant differences were detectable on inspection of either the whole dataset or the dilated cardiomyopathy-associated proteins specifically (**Fig 5C and 5E**).

## Discussion

Bi-allelic loss-of-function mutations were recently reported in *ITPA* in seven affected individuals from four families with early-infantile encephalopathy, a distinctive pattern of white matter disease evident on brain MR imaging, microcephaly and progressive neurological disease[17]. While no measurement of rI/dI incorporation into RNA or DNA was presented from these cases, the clinical and genetic evidence for causation was compelling in this group of children. Here we have shown that a Martsolf-like syndrome with iDCM, is an allelic disorder. There is also evidence of phenotypic overlap between the disorders as one of the seven affected individuals reported by Kevelam et al.[17] had iDCM and three had early onset cataracts. Taken together with the existing mouse genetic data [26, 28], these data strongly support an essential role for ITPase activity in development and maintenance of brain, eye and heart function in mammals.

Since 2015 there have been no further reports of severe ITPase deficiency. The severity, the distinctive phenotype and the increasing use of whole exome sequencing in clinical diagnostics make it unlikely that this would be missed. This suggests that ITPase deficiency is genuinely very rare. In gnomAD (November 2018) there are 57 individuals heterozygous for 25 different loss-of-function *ITPA* alleles. These variants have a combined MAF of 0.0003 indicating a minimum carrier frequency of ~1:1672, which, assuming random mating, would give a minimum expected birth incidence of ~1:11 million for biallelic LOF alleles in *ITPA*. Interestingly, the c.452G>A;p.Trp151Ter variant shows evidence of a founder effect in Finland with a carrier rate of 1 in 1200 but this would still predict a minimum birth prevalence of < 1 in 5 million. This presumably explains why both families we have identified are consanguineous.

**Fig 6:**
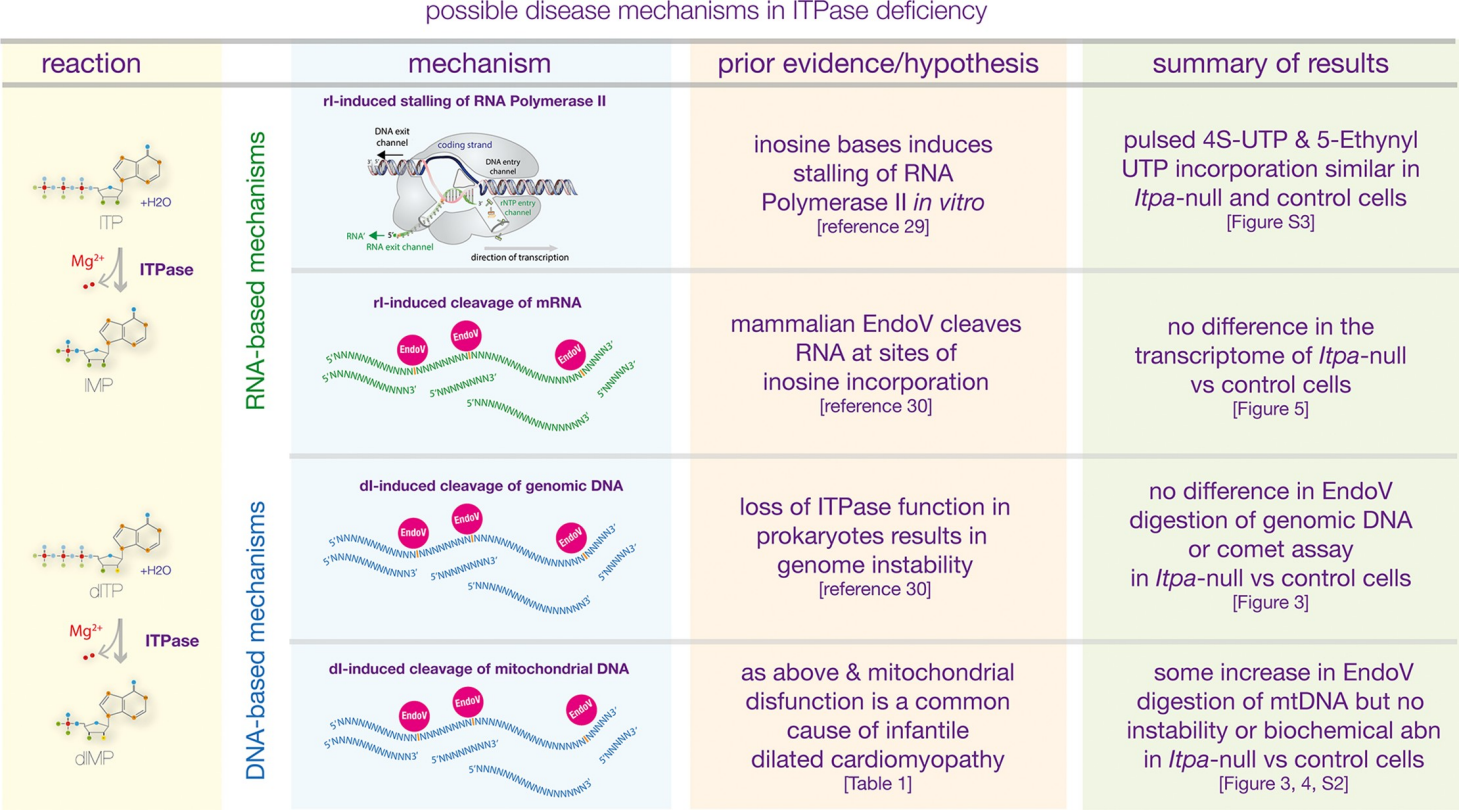
Summary of *ITPA*-related disease mechanisms tested. The left hand panel shows a cartoon of the ITPase reactions involving dITP and ITP to create dIMP and IMP respectively. The top half of the right hand panels summarise RNA-based mechanisms and the bottom half DNA-based mechanisms. The blue panel summarises the molecular basis of the mechanism. The orange panel the prior evidence and the hypothesis and the green panel the data that is presented in this paper that is relevant to each of the hypotheses. Under the text in each of the right hand panels there are square brackets which indicate which reference, table or data figure are relevant to the preceding text.

There are obvious candidate mechanisms for a cellulopathy associated with ITPase null state (summarised in Fig 6). First, instability of the nuclear genome induced by dITP incorporation into DNA (as seen in E coli[27]); second, instability of the mitochondrial genome via the same mechanism; third, inhibition of RNA polymerase II by rI (previously demonstrated *in vitro*[29]); fourth, instability of mature transcripts through EndoV-mediated degradation of rI-enriched mRNA; finally, induction of energy deficiency state due to biochemical perturbation of mitochondrial function. In this paper we have attempted to address each of these and failed to observe any single major effect. The differential incorporation of inosine bases between DNA and RNA is interesting. This may reflect the evolution of efficient DNA surveillance and repair mechanisms to deal with deamination of adenosine bases to form dI with the steady state for inosine in DNA being ~1 per 10^6^ nucleotides[30]. This would suggest that even moderate increases in the incorporation of dITP into DNA in ITPase null cells are likely to be below the limit of detection for the assays used here.

In this regard it is significant that we could detect low-level dI incorporation into the mitochondrial genome. Importantly we could detect no effect on either the quantity or structural integrity of mtDNA from hearts from *Itpa*-null embryos as compared to controls (**S1 Fig**). The lack of evidence for a DNA-based mechanism taken together with the correlation of rI incorporation in RNA with organ severity suggested to us that there may be a transcriptomic mechanism of disease.

Enzymatic A-to-I editing in RNA is used to “recode” specific transcripts in a highly regulated manner[31] and this may explain why there is no rI-induced repair system for RNA. However, human EndoV[30] is capable of cleaving RNA at inosine bases[32, 33] and over-incorporation of rI could lead to a generalized instability of the transcriptome. We found evidence of a reduction in the transcript abundance of some longer mRNA extracted from *Itpa*-null mouse embryonic heart. This effect is difficult to interpret given that it is a relatively minor change and is plausibly a secondary effect of the disease process rather than due to rI-induced RNA instability. One way to address this problem would be to create animals who are null for both *Itpa* and *Endov* and thus determine if loss of the ribonuclease activity would rescue any or all of the *Itpa* phenotypes. Although we have not reported the details here, our preliminary work using *Itpa*/*EndoV* double KO mouse embryos suggested no reduction in rI incorporation into RNA.

It seems probable that the major disease mechanism in severe ITPase deficiency related to either inosine base production or rI incorporation. It is not clear why the heart, brain and developing eye are more sensitive to the perturbation. The modest reductions in RNA levels in the mouse orthologs of two of the known genetic causes of cardiomyopathy in humans (Fig 5G; Ttn and Ryr2) are interesting but difficult to interpret. There no detectable generalised effect on the transcriptome, even for very long transcripts, and thus these reductions in specific transcripts are more likely to represent an early marker of cardiomyocyte disfunction rather than a primary pathogenic mechanism. A major challenge in studying the cellular basis of *ITPA*-associated disease is the large number of possible consequences of altering the composition of the cellular nucleotide pool. These include intracellular signalling, post-translational modification and energy production in addition to those detailed in Fig 6. That fact that *ITPA*-null cells grow at a normal rate with normal morphology may indicate that the perturbation me be individually subtle but collectively have catastrophic consequences in vulnerable tissues such as the brain and heart. A clear understanding of the disease mechanism is important as it may lead to therapies that will ameliorate the progressive cardiac and neurological effects of this rare but important disease.

## Methods and models

### Clinical samples and information

Our cohort consists of DNA samples from 85 families, submitted by referring clinicians for research screening for Warburg Micro syndrome, Martsolf and Martsolf-like syndromes[8]. Affected individuals in this cohort are negative for causative variants in the coding sequences of *RAB3GAP1*, *RAB3GAP2*, *RAB18* and *TBC1D20*, the genes previously associated with these disorders. Informed, written consent has been obtained from both participating families. The consent process and molecular analysis used protocols approved by the Scotland A Multicentre Research Ethics Committee (04:MRE00/19; The Genetic Basis of Brain Growth and Development) in the UK.

The mouse work was done under a UK Government Home Office animal licence: 60/4424. The work was overseen by the University of Edinburgh Animal Welfare and Ethical Review Body (AWERB).

### Whole exome sequencing and analysis

DNAs from Families 4911 and 5196 (nuclear trios) were enriched for exonic sequence using kits indicated in **S1 Table** and sequenced using Illumina HiSeq technology as described previously[34]. Sequence reads were aligned to the GRCh37 human genome reference assembly with BWA mem 0.7.10[35]. Duplicate reads were marked with Picard MarkDuplicates 1.126. Reads were re-aligned around indels and base quality scores re-calibrated with GATK 3.3[36]. Single nucleotide variants and small indels were called with GATK 3.3 HaplotypeCaller on each sample and GenotypeGVCFs to produce a raw variant call set. Variants were annotated using the Ensembl Variant Effect Predictor[37]. Statistics for alignment, read depth and estimated heterozygous SNP detection sensitivity for each individual are listed in **S1 Table**. Sequence variants were filtered using minor allele frequency (MAF) of < 0.001, plausibly deleterious consequence and bi-allelic inheritance. A screen for plausibly disease associated genotypes associated with known developmental disorder genes was performed using the DDG2P pipeline as previously described[21].

### Sanger sequencing

PCR amplification of the coding exons of *ITPA* and intron-exon boundaries was carried out using flanking primers with M13 tags to facilitate later sequencing (see **S2 Table**). Primers were designed using ExonPrimer software on the basis of the reference sequence NM_033453. Sequencing reactions were carried out with BigDye Terminator 3.1 reagents (Applied Biosystems), according to manufacturer's instructions. Sequencing data was analysed with Mutation Surveyor software (SoftGenetics).

### Cell culture

Cells were maintained under 5%CO_2_ at 37°C. Lymphoblastoid cell lines (LCLs) were maintained in suspension in RPMI1640 media (Gibco) supplemented with 10% foetal calf serum, 1mM oxalocacetate, 0.45mM pyruvate, 0.03% glutamine, 1% penicillin/streptomycin and 0.2 I.U/ml insulin. Embryonic stem (ES) cells were maintained in adherent culture in GMEM supplemented with 10% foetal calf serum, 0.1mM non-essential amino acids, 2mM L-Glutamine, 1mM sodium pyruvate and 106 units/L LIF.

### Antibodies and western blotting

Rabbit polyclonal antibody raised to full-length ITPA was obtained from Millipore. Rabbit polyclonal antibody raised to an N-terminal portion of ITPA was obtained from LSBio. Goat polyclonal antibody raised to β-tubulin was obtained from Abcam.

Cells were lysed in a buffer containing 0.5% (v/v) Nonidet P-40 in a solution of 150mM NaCl, 10mM EDTA and 50mM Tris-HCl (pH = 7.5) to which a protease inhibitor cocktail (Roche) was added. Tissue samples were lysed directly in 1x NuPage LDS Sample Buffer (Thermo Fisher) containing 5% β-mercaptoethanol. SDS-PAGE and Western blotting were carried out according to standard methods. ECL 2 Western blotting substrate (Pierce) was used to produce chemiluminescent signal, HyperFilm ECL (General Electric) was developed using a Konica Minolta SRX-101A.

### Detection of inosine bases in hydrolysed RNA and DNA

Cellular RNA and DNA were purified using RNAeasy (Qiagen) and BACC2 (GE Healthcare) kits respectively. mtDNA isolation was performed using a mitochondrial DNA isolation kit (Abcam).

For analysis of nucleic acid composition by mass spectrometry, digestion to single nucleotides was carried out. A combination of either 50μg/ml RNAseA (RNA) or 20U/ml DNAseI (DNA)(Roche Diagnostics) respectively and 80U/ml NucleaseP_1_ (Sigma) was used as previously described[38]. Both purification and digestion was carried out in the presence of a 20μM concentration of the adenosine deaminase (ADA) inhibitor deoxycoformycin (DCF)(Sigma). Digestions were carried out in a buffer containing 1.8mM ZnCl_2_ and 16mM NaOAc, pH = 6.8 at 37°C overnight. Nucleases were then removed with 10,000 MW cut-off spin columns (Amicon). Samples were loaded onto a ZIC-pHILIC column using a Dionex RSLCnano HPLC and the eluate was applied to a Q Exactive mass spectrometer in negative mode. The instrument was operated in tSIM mode and data were quantified using XCalibur 2.0 software.

For analysis of genomic and mitochondrial DNA composition by Endov-digestion and alkaline-gel electrophoresis, DNA samples were treated with 10 U of Endonuclease V (NEB) with the supplied buffer for 2 hours at 37°C. DNA strands were separated by incubation at 55°C in loading buffer containing 3% Ficoll (type 400) and 300mM NaOH. Samples were separated on agarose gels (50mM NaOH, 1mM EDTA) with a solution of 50mM NaOH, 1mM EDTA used as running buffer. After electrophoresis, gels were neutralized and stained with SYBR Gold (Invitrogen).

### Comet assays

Alkaline comet assays were carried out using the Trevigen CometAssay electrophoresis kit according to manufacturer’s instructions. Briefly, cells were embedded into low melting agarose on comet slides and incubated in lysis solution overnight in the dark at 4°C. They were incubated in a solution of 300 mM NaOH, 1 mM EDTA for 30 minutes at room temperature, then electrophoresed in this solution for 30 min at 21 volts at 4°C in the dark. Comet slides were immersed in 70% ethanol for 10 min at room temperature and dried at 37°C for 15 minutes. They were then stained with 1x SYBR gold in TE buffer (pH 7.5) for 30 minutes at room temperature, dried for an additional 15 minutes at 37°C and visualized with a Zeiss Axioskop 2 epifluorescence microscope with a 10x objective. Data were analysed with CaspLab 1.2.3 software.

### Long-range PCR of mitochondrial DNA

10ng DNA samples were amplified using the TaKaRa LA Taq polymerase mix with primers flanking nucleotide positions 272 and 16283 on the mouse mitochondrial genome (NC_005089; see **S2 Table**). A long PCR template program was used as follows: 94°C– 2 minutes, 35 cycles of 94°C– 30 seconds and 65°C– 16 minutes followed by a final extension of 72°C– 16 minutes.

### Quantitative PCR

For quantitative PCR (qPCR) of mitochondrial (mtDNA) and genomic DNA (gDNA), DNA preparations (retaining both species) were made from cell and tissue samples using Viagen reagent (Viagen Biotech) according to manufacturer’s instructions. For analysis of gene expression by qPCR, RNA was extracted using Trizol reagent together with an RNeasy mini kit (Qiagen) according to manufacturer’s instructions. Purified RNA was used immediately as a template for production of cDNA using a First Strand cDNA Synthesis Kit for RT-PCR (AMV) (Roche).

qPCR analysis was carried out on a LightCycler 480 (Roche). Amplification from mtDNA and gDNA was carried out using pairs of primers designed to amplify from the mtCO1 locus of the mitochondrial genome and the *Gapdh* locus of the autosomal genome. Amplification from cDNA was carried out using primers designed to amplify from TTN, RYR2, TNNT2 and GAPDH cDNAs. Amplification from NKX2-5 was carried out using commercial TaqMan probes (Mm01309813_s1_Nkx2-5). PCR amplification with unlabelled primers was quantified through binding of specific mono color hydrolysis probes (Roche). Data were analyzed using LightCycler 480 software version 1.5.0 (SP4) (Roche). Primers were designed using the Universal ProbeLibrary Assay Design Center and are listed in **S2 Table**.

Droplet Digital PCR (ddPCR) reactions were carried out according to manufacturer's instructions (Biorad). In each reaction, cDNAs were combined with a VIC-labeled TaqMan control probe, mouse GAPD or eukaryotic 18S (Life Technologies). Primers specific for target genes are as above. Droplets were generated using a Biorad QX200 or QX200AutoDG droplet generator, PCRs were carried out using a C1000 Touch Thermal Cycler, and droplets were analyzed on a QX100 Droplet Reader. The data were analyzed using Quantasoft software (QuantaLife).

### Mitochondrial enzyme histochemistry

Tissue samples from Itpa-null embryos and littermate controls (e16.5-e18.5) were frozen in liquid nitrogen-cooled isopentane prior to sectioning. Cryostat sections were stained for individual activities of COX and SDH and also for sequential COX/SDH activity. Briefly, sections were reacted for 45 min at 37°C with COX reaction media (4 mM diaminobenzidine tetrahydrochloride, 100 μM, cytochrome *c* and 20 μg/ml catalase in 0.2 M phosphate buffer, pH 7.0) and 40 min at 37°C with SDH media (1.5 mM nitroblue tetrazolium, 1 mM sodium azide, 200 μM phenazine methosulphate, 130 mM sodium succinate, in 0.2 M phosphate buffer, pH 7.0).

### Microarray

RNA was extracted from e16.5 mouse hearts using Trizol reagent together with an RNeasy mini kit (Qiagen) according to manufacturer’s instructions. RNA quality was assessed using an Agilent Bioanalyser instrument and Total RNA nano. RNA integrity numbers (RIN) were ≥9.1 for all samples. Transcriptome analysis was carried out by Aros Applied Biotechnology A/S using the Affymetrix MTA1.0 microarray. Data were analysed using Affymetrix Transcriptome Analysis Console 3.0 and custom R scripts.

### Label-free quantitative proteomics

Protein was extracted from embryonic mouse hearts in a buffer containing 8M Urea, 75mM NaCl and 50mM Tris, pH = 8.4 by sonication at 0–4°C in a Bioruptor device (Diagenode) together with silica beads. Protein concentrations were quantified using a BCA assay (Pierce) and then 100μg of each sample was subjected to in-solution tryptic digest. Samples were loaded onto a C18 column using a Dionex RSLC Nano HPLC and the eluate was applied to a Q Exactive mass spectrometer. The data were quantified using XCalibur 2.0 software.

### Generation of Itpa-null mouse ES cells and embryos

*Itpa*-null mouse ES cells were generated using CRISPR/Cas9 genome editing [24, 25]. Paired guide RNA (gRNA) sequences were selected using the online CRISPR design tool (http://crispr.mit.edu/). Oligonucleotides encoding these sequences (**S2 Table**) were annealed and ligated into pX461 and pX462 plasmids (Addgene). Recombinant plasmids were verified by direct sequencing. For each targeted locus, the E14 ES cells were transduced with 1μg of each vector using the Neon system (Life Technologies) according to manufacturer's instructions. Cells were allowed to recover for 24 hours, then treated for 24h with 1 μg ml^−1^ puromycin in order to select for cells containing the px462 construct. To select single cells also containing the px461 construct, fluorescence activated cell sorting into 96-well plates was carried out using a FACSJazz instrument (BD Biosciences). Clonal cell lines were analysed by direct sequencing of targeted alleles and by Western blotting. Sequencing primers are shown in **S2 Table**. To facilitate sequence analysis, PCR products were cloned into pENTR/D-TOPO vectors prior to sequencing.

Cytoplasmic zygotic injection of wild-type Cas9 mRNA together with *in vitro* transcribed gRNAs was used to generate *Itpa*-null mouse embryos. This approach was also used to produce heterozygous-null animals used to establish transgenic mouse lines. The plasmid vectors described above were used as a template for PCR amplification together with forward primers incorporating T7 promoter sequences and a universal reverse primer (see **S2 Table**). RNA was synthesised using a HiScribe T7 High Yield RNA Synthesis Kit (New England Biolabs) according to manufacturer’s instructions. DNA preparations were made from tissue samples using Viagen reagent (Viagen Biotech). Genotyping was carried out by direct sequencing of targeted alleles and by Western blotting as above. Following initial genotyping of *Itpa*-null animals produced by crossing heterozygous-null founders, subsequent genotyping of transgenic lines was conducted through PCR analysis.

### Optical projection tomography and morphometry

E16.5 mouse embryos were mounted in 1% agarose, dehydrated in methanol and then cleared overnight in a solution containing 1 part Benzyl Alcohol and 2 parts Benzyl Benzoate. Imaging was conducted with a Bioptonics OPT Scanner 3001 (Bioptonics, UK) using brightfield analysis to detect tissue autofluorescence for capture of anatomical and signal data (wavelengths: excitation at 425 nm, emission: 475 nm). The resulting data were reconstructed using Bioptonics proprietary software (Bioptonics, MRC Technology, Edinburgh, UK), automatically thresholded to remove background signal, then merged into a single 3D image output using Bioptonics Viewer software. Measurements of internal chest cavity diameter, maximum heart diameter, cardiac wall cross-sectional area and total heart cross-sectional area were taken for five embryos per genotype.

## Supporting information

S1 Table Documents the technical metrics relating to the trio whole exome sequencing on families 4911 and 5196. (XLSX)

S2 Table Lists the oligonucleotide primers used for; i) sequencing the candidate human genes (including ITPA); ii) creation of genome editing reagents; iii) sequencing mouse *Itpa;* iv) mitochondrial genome analysis; v) quantitative RTPCR. (XLSX)

S1 Fig ITPA-deficient models. (A) Western blotting of the ITPA-deficient mouse embryonic stem cell (ES) cell line characterized in Fig 3. ITPA protein is detected in lysates from control but not *Itpa*-null cells upon probing the blot with polyclonal antibodies raised to full-length ITPA (Millipore) and an N-terminal domain of the protein encoded by sequence 5’ of that mutated by CRISPR (LSBio). Blotting for Tubulin serves as a loading control and each lane on the blot corresponds to an individual lysate sample. Cloning of the targeted region and subsequent direct sequencing identified c.-33_125_129delinsCTGTCTGTTT and c.-33_125_147del alleles. (B) Western blotting of limb lysates from the embryos shown in OPT analysis below. ITPA protein is detected in lysates from control but not *Itpa*-null lysate upon probing the blot with the antibody to full-length ITPA. Blotting for Tubulin serves as a loading control. Direct sequencing identified c.258dupC and c.234_257del alleles. Nucleotide numbering reflects cDNA numbering with +1 corresponding to the A of the ATG translation initiation codon in the reference sequence NM_025922. (C-E) Representative images from optical projection tomography (OPT) of wild-type, heterozygote and *Itpa*-null e16.5 embryos. (F-H) Comparative morphometry from OPT of control and *Itpa*-null e16.5 embryos.
(DOCX)

S2 Fig Sequencing of mtDNA in control and *Itpa*-null tissues and metabolic analysis of mitochondrial function in control and *Itpa*-null ES cells. (A) Quantitative RT-PCR (qPCR) on total DNA shows that ratios of mtDNA to genomic DNA are comparable between control and *Itpa*-null tissues. The data shown are derived from analysis of a minimum of three individual DNA preparations per genotype, per tissue, each analysed in triplicate. (B) Long-range PCR (LR-PCR) of the mitochondrial genome from control and *Itpa*-null heart shows no evidence for increased deletions in the *Itpa*-null heart. The data shown are representative of three independent experiments. (C) Ion Torrent sequencing shows no evidence for increased base substitutions in mitochondrial genomes in *Itpa*-null kidney and heart as compared to controls. (D) MinION sequencing shows no evidence for increased deletions in mitochondrial genomes in *Itpa*-null kidney and heart as compared to controls. The primers used in A-D are listed in S2 Table (E) Metabolic tracer analysis on the ES cells using ^13^C_5_-glutamine shows no evidence for substantially altered metabolism in *Itpa*-null ES cells as compared to controls.
(DOCX)

S3 Fig Transcription rate and transcript stability in control and *Itpa*-null ES cells. (A) Quantitation of 4-thiouridine (4sU) incorporation into RNA following 30 minutes treatment. Total RNA was purified from control and *Itpa*-null mouse ES cells following 0, 2, 8 and 24 hours recovery. Thiol was biotinylated then levels of biotin in repurified RNA was measured using a fluorescence-based assay. Data are combined from three independent experiments (n = 7). (B) Quantitation of RNA containing 4-thiouridine (4sU) following 30 minutes treatment. Total RNA was purified from control and *Itpa*-null mouse cells following 0, 2, 8 and 24 hours recovery. Thiol was biotinylated and then biotinylated RNA was purified using magnetic streptavidin beads. Data are expressed as a ratio of purified biotinylated RNA to total RNA (n = 5). (C) Quantitation of 5-Ethynyl Uridine (EU) incorporation into RNA following 15 minutes treatment. Levels of incorporation were determined by fluorescence-based assay and FACS analysis. Data points are derived from geometric mean fluorescence of at least 1000 cells for each replicate sample (n = 4). Dashed lines in A and C indicate background readings determined through parallel analyses of untreated cells.
(DOCX)

S1 Materials and Methods This text describes the materials and methods used to generate the data presented in the supplementary tables and figures. (DOCX)
