# Supplementary material for: ITPase deficiency causes a Martsolf-like syndrome with a lethal infantile dilated cardiomyopathy
Source: PLoS Genet. 2019 Mar 11;15(3):e1007605. doi: 10.1371/journal.pgen.1007605 (PMC6428344; doi:10.1371/journal.pgen.1007605)
Supplement: S1 Materials and Methods — (DOCX) [file pgen.1007605.s006.docx]

**Supplementary Methods and Models**

**Optical projection tomography and morphometry**

E16.5 mouse embryos were mounted in 1% agarose, dehydrated in methanol and then cleared overnight in a solution containing 1 part Benzyl Alcohol and 2 parts Benzyl Benzoate. Imaging was conducted with a Bioptonics OPT Scanner 3001 (Bioptonics, UK) using brightfield analysis to detect tissue autofluorescence for capture of anatomical and signal data (wavelengths: excitation at 425 nm, emission: 475 nm). The resulting data were reconstructed using Bioptonics proprietary software (Bioptonics, MRC Technology, Edinburgh, UK), automatically thresholded to remove background signal, then merged into a single 3D image output using Bioptonics Viewer software. Measurements of internal chest cavity diameter, maximum heart diameter, cardiac wall cross-sectional area and total heart cross-sectional area were taken for five embryos per genotype.

**Ion Proton sequencing**

mtDNA was amplified from wt and ITPA null embryonic heart tissue using long-range PCR as described in Methods and Models. ~16kb PCR products were purified using gel-purification columns (Qiagen) then sheared using a Covaris system. Sheared DNA was largely 150-300bp as assayed by Bioanalyzer. Library preparation and barcoding was carried out using ClaSeek Library Preparation and Ion Torrent Ion Xpress 1-96 kits respectively, according to manufacturer’s instructions. Pooled DNAs were sequenced on the Ion Torrent platform (Invitrogen). Three biological replicates were analysed, per tissue, per genotype.

Unpaired reads were aligned to the mm10 chrM reference sequence using bowtie (v2.1.0) and samtools (v1.2). BAM files were generated incorporating only reads with high mapping quality (MAPQ>30). Substitutions were called only when base quality (BQ) score ≥20, and where they were represented at least once in sequence from both forward and reverse strands.

**MinION sequencing**

mtDNA was amplified from wt and ITPA null embryonic heart tissue using long-range PCR as described in Methods and Models. ~16kb PCR products were purified using gel-purification columns (Qiagen). Library preparation and sequencing was carried out according to a modified Nanopore Sequencing Kit protocol SQK-MAP006. NEBNext End Repair, dA-Tailing Module and BCA01-12 PCR barcoding kits were used to prepare the DNAs prior to pooling. Pooled DNAs were sequenced on the MinION platform (Oxford Nanopre) in two 20-hour runs. Three biological replicates were analysed, per tissue, per genotype.

FAST5 output from the sequencing platform was converted to FASTQ then FASTA with pore [1] and fastx (v0.0.13). High-quality 2D reads were aligned to the mm10 chrM reference sequence with LAST (v704). Indexed BAM files were generated with samtools (v1.2). Deletion percentage for each read was calculated as (deleted bases/aligned bases)x100, then mean percentages calculated for each replicate.

**Metabolic tracer experiments**

Mouse ES cells on 10cm^3^ dishes were treated with 300µg/ml (2mM) ^13^C_5_-glutamine in full media (GMEM supplemented with 10% foetal calf serum, 0.1mM non-essential amino acids, 1mM sodium pyruvate and 106 units/L LIF) under 5%CO2 at 37°C. Following washing x2 in ice-cold PBS, metabolites were extracted in extraction buffer containing 50:30:20 methanol/acetonitrile/water. Samples were loaded onto a ZIC-pHILIC column using a Dionex RSLCnano HPLC and the eluate was applied to a Q Exactive mass spectrometer in wide scan negative mode. Data were quantified using XCalibur 2.0 software. Samples were produced and analysed in triplicate.

**4-thiouridine incorporation assays**

Mouse ES cells on 10cm^3^ dishes were grown to 70% confluence, and then treated with 100µM 4-thiouridine (4sU) in full media (GMEM supplemented with 10% foetal calf serum, 0.1mM non-essential amino acids, 2mM L-Glutamine, 1mM sodium pyruvate and 106 units/L LIF) for 30 minutes at 37°C. Cells were washed and then allowed to recover for 0, 2, 8 and 24 hours in full media. Following recovery, cells were again washed and then lysed in Qiazol reagent (Qiagen). RNA was purified from the resulting lysates using a miRNeasy kit (Qiagen) according to manufacturer’s instructions. Total RNA was quantified using a Nanodrop 1000 instrument, then equal quantities of the RNA samples in each experiment were subjected to biotinylation by rotation for 2 hours with 20mg/ml EZ-link biotin HPDP (Thermo Scientific) in a buffer containing 40% dimethylformamide, 1mM EDTA and 10mM Tris, pH7.4. RNA was re-purified with chloroform/isoamyl alcohol (24:1). Excess biotin HPDP was removed by centrifugation in MaXtract high-density tubes and RNA was precipitated from the aqueous phase using glygogen, NaCl and isopropanol. Pellets were washed with 75% EtOH, resuspended in nuclease-free water and RNA concentrations quantified.

For quantification of biotin, 20µg RNA from each sample was analysed. A FluoReporter kit (Molecular Probes) was used together with a plate reader according to manufacturer’s instructions. For quantification of biotinylated RNA, biotinylated RNA was first purified from 40µg total RNA using MyOne streptavidin T1 magnetic beads (Life Technologies). Beads were first washed with a solution of 100mM NaOH and 100mM NaCl and then with 100mMNaCl to remove trace nucleases. Biotinylated RNA was bound by rotation for 15 minutes in a solution containing 1M NaCl, 0.5mM EDTA 0.05% v/v Tween 20 and Tris pH7.5., then washed three times in the same buffer. It was eluted with 100mM DTT and then purified using an RNeasy MinElute clean-up kit (Qiagen). RNA concentration was determined using a Qubit flurometer (Life Technologies) together with RNA high sensitivity assay reagents according to manufacturer’s instructions.

**5-Ethynyl Uridine incorporation experiments**

Mouse ES cells on 10cm^3^ dishes were grown to 70% confluence, and then treated with 1mM 5-Ethynyl Uridine (EU) in full media (GMEM supplemented with 10% foetal calf serum, 0.1mM non-essential amino acids, 2mM L-Glutamine, 1mM sodium pyruvate and 106 units/L LIF) for 15 minutes at 37°C. Cells were washed and then immediately trypsinised, pelleted, washed again and finally resuspended in a fixation buffer containing 4% paraformaldehyde in PBS. EU incorporated into RNA was labelled with Alexa Fluor 488 using a Click-iT RNA HCS Assay kit (Molecular Probes). The associated protocol was adapted for FACS analyses. Briefly, cells were permeablized with 0.5% Triton-X-100 in PBS for 15 minutes at room temperature, then the permeablization buffer was removed by centrifugation. Cells were stained by resuspension in ‘Click-iT reaction cocktail’ and incubation for 30 minutes at room temperature in the dark. They were then washed once in ‘Click-iT rinse buffer’ and twice in PBS by centrifugation before being subjected to analysis on a BD FACSAriaII instrument. Data were analysed using BD FACSDiva 8.0.1 software.

**Supplementary references**

[1] [poRe ref] “poRe : an R package for the visualization and analysis of nanopore sequencing data. / Watson, Mick; Thomson, Marian; Risse, Judith; Talbot, Richard; Santoyo-Lopez, Javier; Gharbi, Karim; Blaxter, Mark.

In: Bioinformatics, Vol. 31, No. 1, 01.2015, p. 114-115.”
