# Supplementary material for: ITPase deficiency causes a Martsolf-like syndrome with a lethal infantile dilated cardiomyopathy
Source: PLoS Genet. 2019 Mar 11;15(3):e1007605. doi: 10.1371/journal.pgen.1007605 (PMC6428344; doi:10.1371/journal.pgen.1007605)
Supplement: Supplementary file 4 [file pgen.1007605.s003.docx]

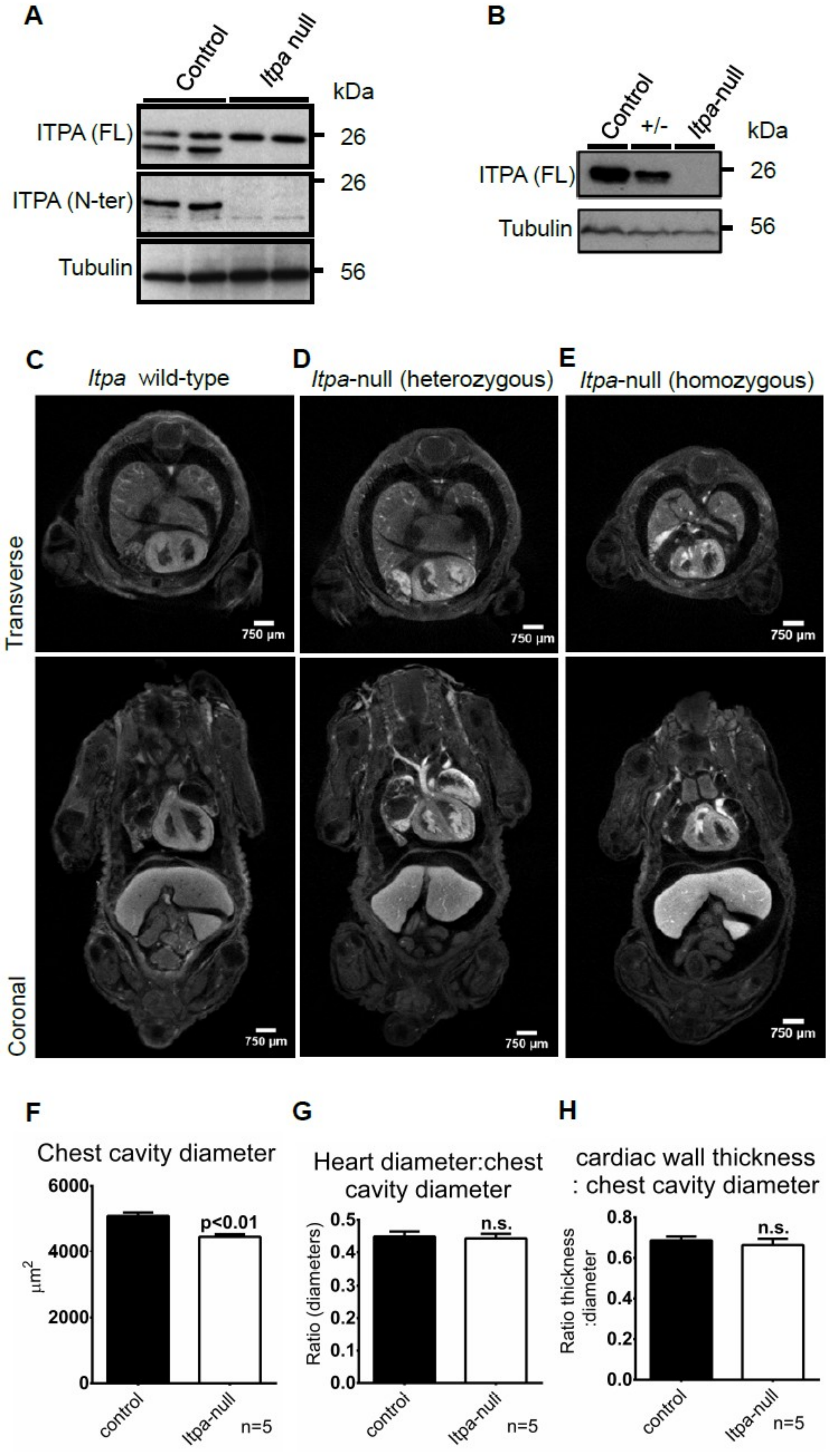

A
Control
Itpa null
kDa
ITPA (FL)
26
ITPA (N-ter)
26
Tubulin
56
B
Control
+/-
Itpa-null
kDa
ITPA (FL)
26
Tubulin
56
C
Itpa wild-type
D
Itpa-null (heterozygous)
E
Itpa-null (homozygous)
Transverse
Coronal
750 µm
F
Chest cavity diameter
µm2
p<0.01
control
Itpa-null
n=5
G
Heart diameter:chest cavity diameter
Ratio (diameters)
n.s.
control
Itpa-null
n=5
H
cardiac wall thickness : chest cavity diameter
Ratio thickness :diameter
n.s.
control
Itpa-null
n=5
