# Supplementary material for: ITPase deficiency causes a Martsolf-like syndrome with a lethal infantile dilated cardiomyopathy
Source: PLoS Genet. 2019 Mar 11;15(3):e1007605. doi: 10.1371/journal.pgen.1007605 (PMC6428344; doi:10.1371/journal.pgen.1007605)
Supplement: Supplementary file 5 [file pgen.1007605.s004.docx]

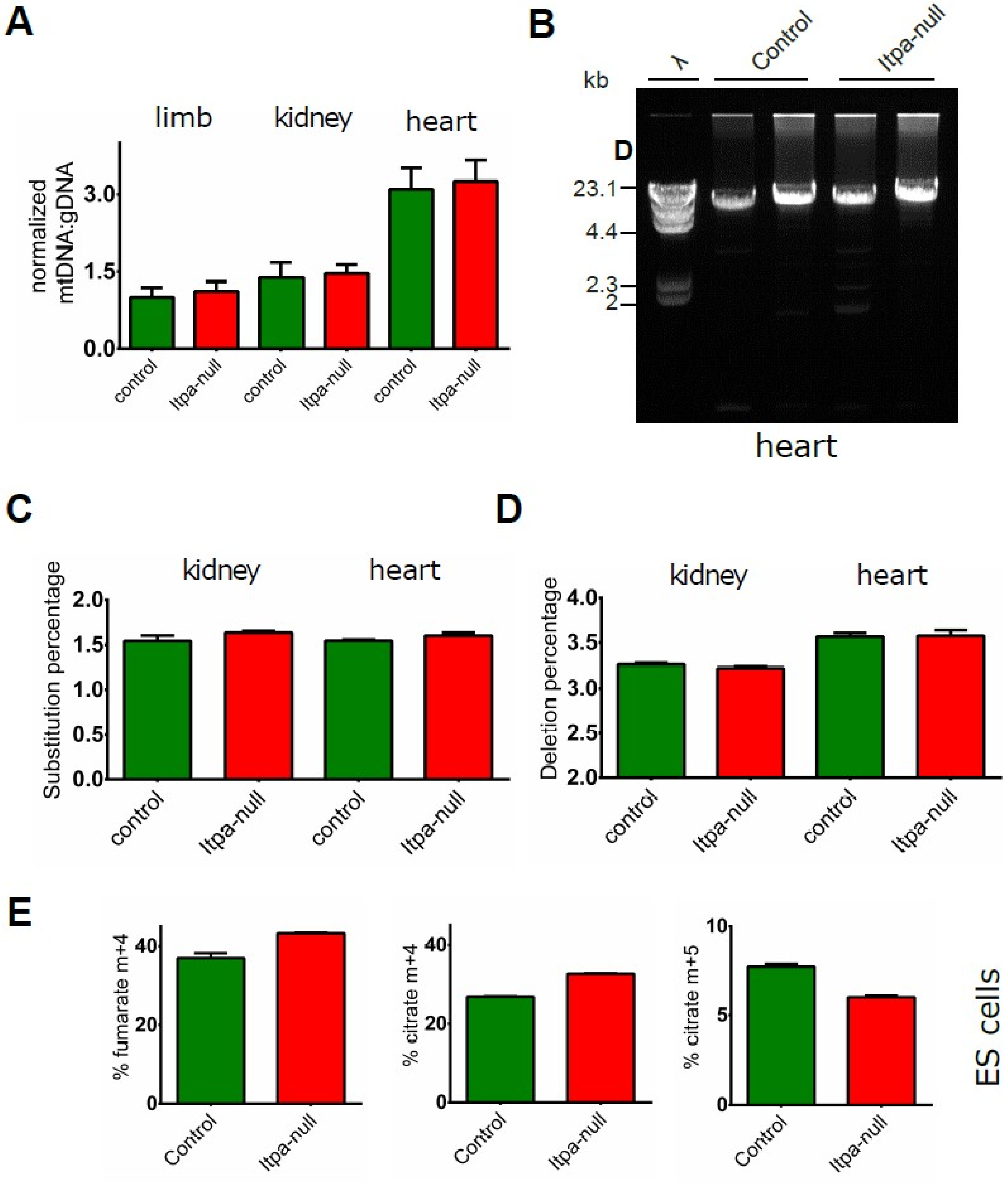
A
limb
kidney
heart
normalized mtDNA:gDNA
0.0
1.5
3.0
control
Itpa-null
B
kb
λ
Control
Itpa-null
D
23.1
4.4
2.3
2
heart
C
kidney
heart
Substitution percentage
0.0
0.5
1.0
1.5
2.0
D
Deletion percentage
2.0
2.5
3.0
3.5
4.0
E
% fumarate m+4
% citrate m+4
% citrate m+5
0
20
40
5
10
Control
ES cells
