# Supplementary material for: ITPase deficiency causes a Martsolf-like syndrome with a lethal infantile dilated cardiomyopathy
Source: PLoS Genet. 2019 Mar 11;15(3):e1007605. doi: 10.1371/journal.pgen.1007605 (PMC6428344; doi:10.1371/journal.pgen.1007605)

Transcription rate and transcript stability

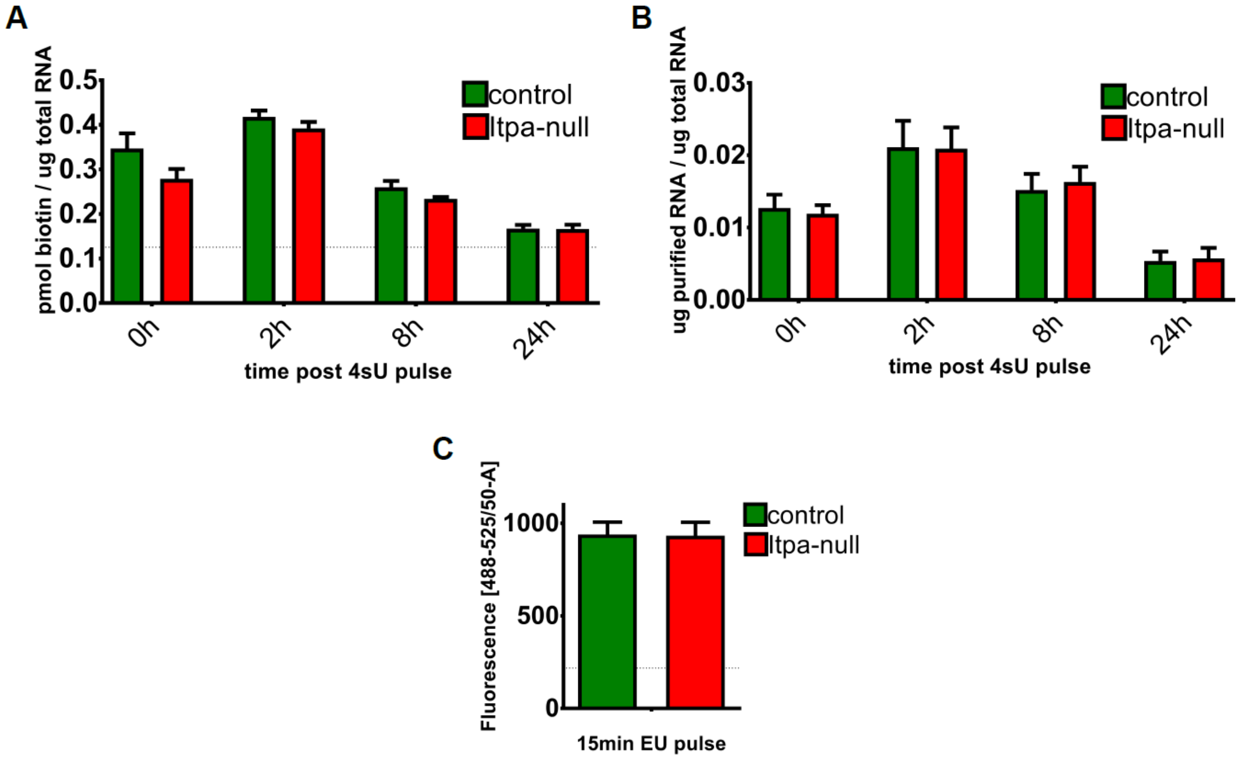

Supplement: Supplementary file 6 [file pgen.1007605.s005.docx]
